# Spinal dural and epidural arteriovenous fistula: Recurrence rate after surgical and endovascular treatment

**DOI:** 10.3389/fsurg.2023.1148968

**Published:** 2023-04-04

**Authors:** Giovanni Giulio Vercelli, Massimiliano Minardi, Mauro Bergui, Francesco Zenga, Diego Garbossa, Fabio Cofano

**Affiliations:** ^1^ Department of Neurosurgery, Ospedale San Giovanni Bosco, Turin, Italy; ^2^ Department of Neurosurgery, University of Turin, Turin, Italy; ^3^ Unità di neuroradiologia, A.O.U. Citta della Salute e della Scienza di Torino, Turin, Italy; ^4^ Dipartimento di Neuroscienze e Salute Mentale, Azienda Ospedaliera Universitaria Città della Salute e della Scienza di Torino, Torino, Italy

**Keywords:** spinal fistula, spinal AVMs, endovascular treatment, epidural fistula, spinal neurosurgery

## Abstract

**Introduction:**

Spinal dural arteriovenous fistula consist of an heterogenous group of vascular malformation often causing severe neurological deficit due to progressive myelopathy. This type of malformation could be associated with subarachnoid or subdural hemorrhage inside the spinal canal. In the English literature surgical treatment is regarded as the best option if compared to endovascular procedure, being the latter associated with an increased risk of relapse despite its less invasiveness.

**Methods:**

In this study a retrospective analysis of 30 patients with spinal dural and epidural fistula associated with perimedullary venous congestion was undertaken. The radiological and clinical presentation of each patient is analyzed, and the grade of myelopathy is classified through the mJOA score.

**Results:**

A total number of 31 out of 41 collected procedures (22 surgery vs. 19 endovascular) were dural fistulas while the remaining 10 were classified as epidural. A 46% recurrence rate for endovascular treatment against 0% for surgical (*p*-value 0.004) was described for dural fistulas, while in the epidural fistula group the rate of recurrence was 80% and 20% respectively for endovascular and surgery treatment (*p*-value 0.6).

**Discussion:**

According to the results, surgical treatment could be considered as first-line treatment for spinal dural arteriovenous fistulas. Endovascular embolization can be proposed in selected cases, as a less invasive technique, for elderly patients or with important comorbidities. In spinal epidural arteriovenous fistulas, in view of the greater invasiveness of the surgical treatment and the non-significant difference in terms of recurrence risk between the two techniques, endovascular treatment could be proposed as a first choice treatment; in the event of a recurrence, a surgical intervention will instead be proposed in a short time.

## Introduction

Spinal arteriovenous malformations account for 3%–4% of all space-occupying lesions (1) of the intradural spinal cord and for 50%–85% of all spinal vascular lesions (2). These malformations include both direct arteriovenous fistulas and true arteriovenous malformations with the presence of a nidus within the spinal cord parenchyma. Dural arteriovenous fistulas (DAVF) account for 70% of spinal arteriovenous shunts. Numerous classifications have been proposed based on the anatomical and pathophysiological characteristics of these lesions (3, 4). It is of paramount importance the distinction in vascular architecture between dural and extradural fistulas. In 2002 and later in 2006, Spetzler et al. (4) modified the previous classification of arteriovenous malformations. In addition to neoplastic vascular lesion, spinal aneurysms, arteriovenous lesions have been classified as extradural arteriovenous fistulas, intradural arteriovenous fistulas (further subdivided into dorsal and ventral), intradural-extradural arteriovenous malformation, intramedullary arteriovenous malformation, and conus arteriovenous malformation.

DAVFs are usually located in the intervertebral conjugation foramen and in the thickness of the dura mater (5): the arterial support is given by a posterior radiculomeningeal branch of the corresponding root segmental artery. The venous drainage of the fistula is given by a radicular vein, a branch of a radiculo-medullary vein, which merges with the peri-medullary venous plexus retrogradely. The venous outflow through the medullary vein and venous plexus are located on the dorsal aspect of the spinal cord in 90% of cases.

Extradural arteriovenous fistulas (EAVFs) are rare types of spinal arteriovenous fistula consisting in a direct connection between a distal branch of the spinal artery and the epidural venous plexus (6, 7). This vascular engorgement causes a large mass effect on the nerve root or spinal cord with symptoms and signs depending on venous congestion and, due to their high-flow nature, on the phenomenon of vascular steal and ischemia.

The identifications of DAVF o EAVF are common with Magnetic Resonance Angiography (MRA) that usually shows Flow voids surrounding the spinal cord corresponding to congested coronal venous plexus with good sensitivity (8). In EAVFs the arterovenous shunt is in the epidural space and only secondarily the intradural venous plexus can be involved, while in DAVFs the arteriovenous shunt is located within the dural sheath of the nerve root and drains directly into an intradural vein without filling of the epidural space (9).

The aim of our study is to compare the recurrence rate of surgical vs. endovascular treatment for DAVFs and EAVFs suggesting then a decision-making algorithm.

## Materials and methods

### Population of the study

This is a retrospective study including consecutive patients with a diagnosis of spinal dural or epidural arteriovenous fistula [according to the Spetzler modified classification (4)] undergoing surgical and/or endovascular treatment from April 2014 to September 2019 at the Neurosurgery and Interventional Neuroradiology units of authors' Institution.

Data were extracted from a database of patients operated for for DAVFs and EAVFs and included demographics, fistula type (dural or epidural), fistula location (cervical, thoracic, lumbosacral), presence and evolution of symptoms (incidental finding, radiculopathy, myelopathy) during follow-up, duration of symptoms prior to diagnosis, type of treatment (endovascular or surgical), adjusted mJOA score to stratify according to myelopathy severity, accurate closure or recurrence of fistula after radiological follow-up examinations with MRA and Spinal Angiography. The selection of patient for surgical or endovascular treatment was undertaken after multidisciplinary evaluation involving neurosurgeons and neuroradiologists. The small sample size does not allow a statistical subdivision of patients by comorbility, age, localization of the malformation, pre- and post-operative clinical status.

### Neuroimaging

**Figure 1:**
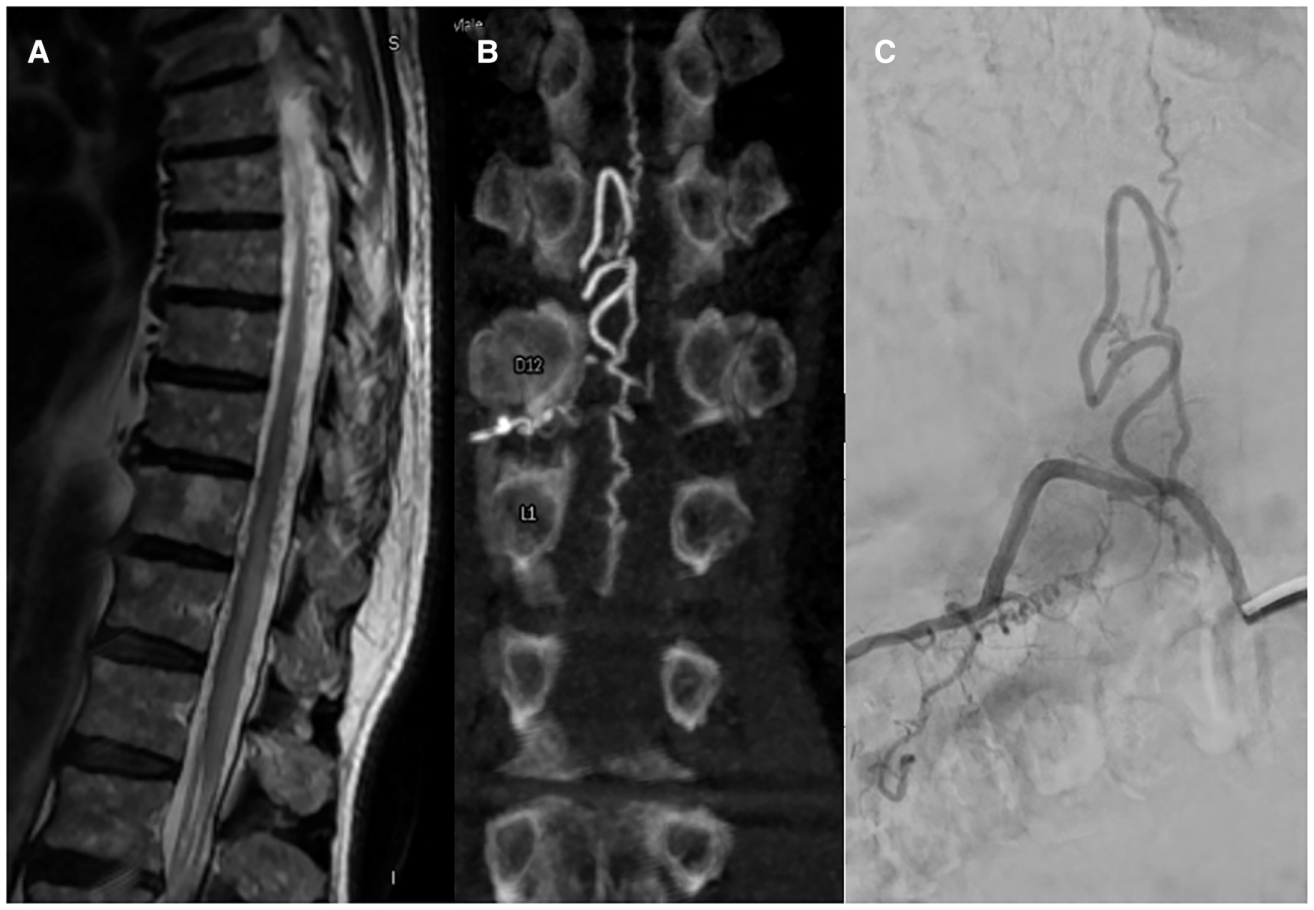
Right D12 dural arteriovenous fistula (**A**) MR; (**B**) VasoCT; (**C**) DSA.

MRA imaging was performed with a 1.5T MRI scanner (Signa Excite HDxt, GE Healthcare, Milwaukee, WI, United States) with an 8-channel spinal coil. Our protocol consisted in a whole spine examination with T1 and T2 sequences, followed by a multiphase 3D angio RM TRICKS sequence (Time-Resolved Imaging with Contrast Kinetick). Spinal angiography was performed with a biplane neuroangiographic system (Allura Clarity, Philips Healthcare, Best, The Netherlands) *via* transfemoral approach under local anesthesia and 4F or 5F catheter. The operators studied the feeders' arteries which supply the fistula based on the results of the MR Angio (Figure 1).

**Figure 2:**
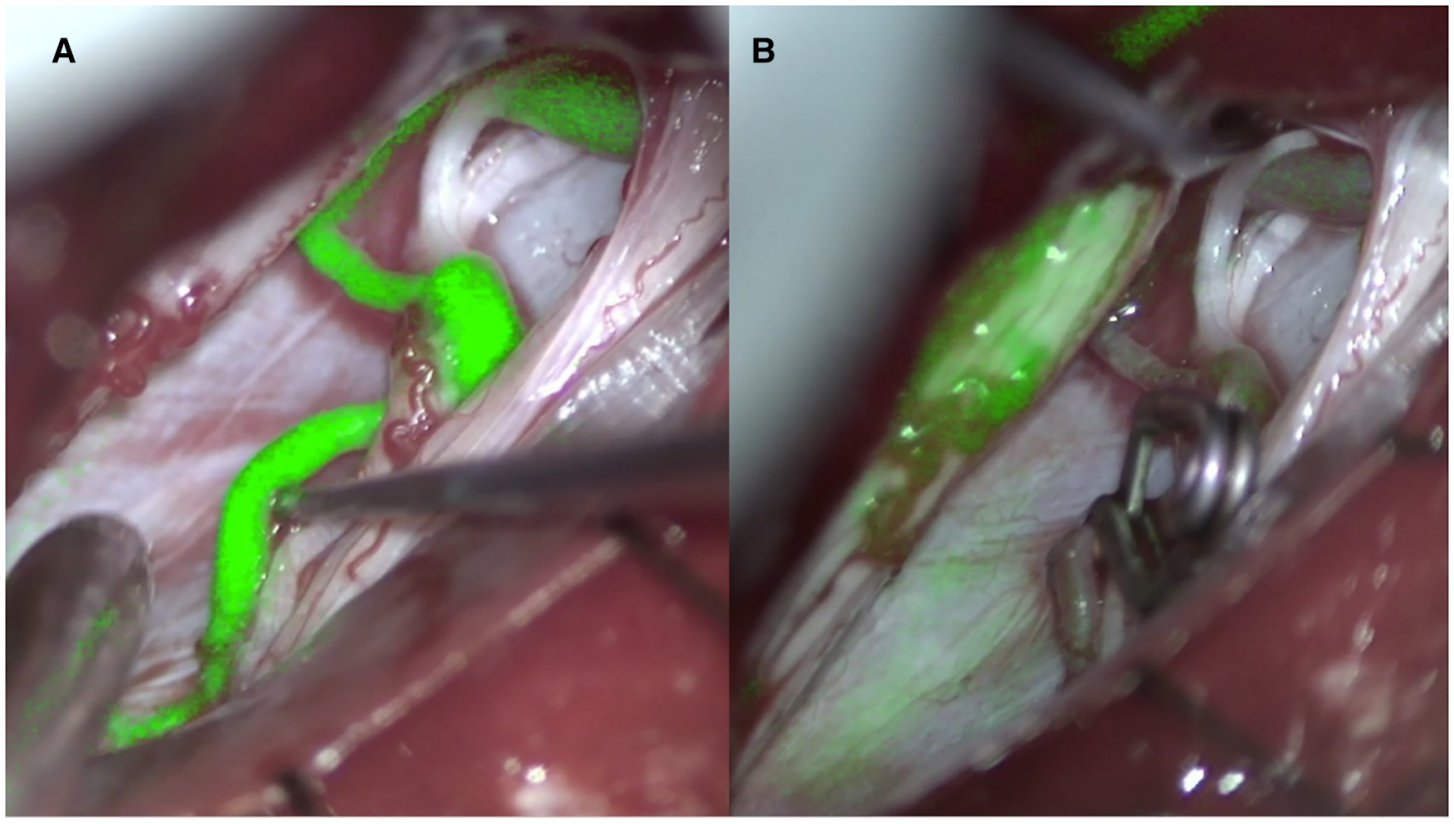
Indocyanine angiography pre (**A**) and post (**B**) clipping of the point of fistula (right D12 dural arteriovenous fistula).

The arterialized posterior median vein is sometimes visible as a serpentine flow void signal on T2-heavy MRI scans. Similar flow voids surrounding the spinal cord correspond to the congested coronal venous plexus. The venous hypertension described above causes spinal cord edema and hyperintensity on T2. The presence of medullary contrastographic impregnation in the affected region is a sign of venous infarction and an unfavorable prognostic indicator for the recovery of neurological function.

Spinal angiography provides the greatest detail on the vascular anatomy of the spinal cord. The shunt can be identified by locating the site of direct transit of contrast from a radiculo-meningeal artery into a dilated radiculo-medullary vein and subsequently into the perimedullary venous plexus located along the dorsal surface of the spinal cord. Adjacent levels should be studied to identify any additional malformed afferent vessels, which may arise anywhere from the vertebral arteries to the sacral artery. Additionally, Adamkiewicz's artery must be identified to plan a safe treatment approach. As suggested by Brinjikji et al. (10) dural and epidural spinal arteriovenous fistula share clinical and radiological findings including hyperintense T2 cord signal, and perimedullary flow-voids. The peculiarity of epidural fistulas consists of appearance on spinal MRI and DSA with a pouch of epidural contrast.

### Clinical outcomes

Clinical information was obtained at the 3-month post-treatment follow-up clinic visit. Healing or persistence of the fistula after treatment was assessed by comparing data between similar imaging modalities at least 1 month after treatment. The main outcomes measured were: (1) the fistula recurrence rate according to the type of fistula (dural or epidural) and the type of treatment (surgical or endovascular); (2) the evolution of symptoms (stability, improvement, or worsening) at follow up.

### mJOA score adapted to evaluate myelopathy

The mJOA score (11) was validated for the evaluation of patients with myelopathy caused by degenerative pathologies of the cervical spine. Considering that in most patients' spinal arteriovenous fistulas are located in thoracic and lumbosacral spine, we modified the mJOA score by eliminating the sections concerning the motor and sensory functions of the upper limbs. Our score therefore considered sphincter dysfunction (which is assigned a score from 0 to 3) and motor dysfunction of the lower limbs (which is assigned a score from 0 to 7). We then stratified our patients into 3 groups: mild myelopathy (8–9 points), moderate myelopathy (6–7 points), and severe myelopathy (0–5 points).

### Statistical analysis

Descriptive statistics were reported as mean or median and standard deviation for continuous variables or frequency and percentage (with 95% CI) for categorical variables, respectively. Proportion comparisons were made with 2-tailed Student's t-test for continuous data and Fisher's exact test for categorical variables. Statistical significance was defined as a *p*-value <0.05. All statistical analyzes were performed using Microsoft Office Excel.

## Results

A total number of 28 patients and 39 surgical or endovascular procedures was included into this study (Table 1). The majority of them was affected by DAVFs (23) with 5 EAVFs recorded. The site of the fistula was cervical for 2 patients, thoracic for 18 patients and lumbosacral for 8 patients. In 3 cases the diagnosis was an occasional finding during tests carried out for other reasons. A radiculopathy was the main symptom for 2 patients while 23 patients had signs and symptoms of myelopathy. Patient age in the study population was in a median of 65 years and a mean of 66 years (standard deviation 7.4). Symptom duration had a median of 6 months and a mean of 8 months (standard deviation 6.9). According to myelopathy severity (assessed by the adjusted mJOA score) 5 patients had no symptoms of myelopathy, 13 patients had mild myelopathy, 6 patients had moderate myelopathy, and 4 patients had severe myelopathy.

**Table 1:** Patient's features.

**Patients**	28
Dural AVF	23
Epidural AVF	5
**Location**	
Cervical	2
Thoracic	18
Lumbosacral	8
**Age (years)**	
Median	65
Mean (St. Dev.)	66 (7, 4)
**Onset clinical symptoms (months)**	
Median	6
Mean (st. dev.)	8 (6, 9)
**Clinical presentation**	
Incidental	3
Radiculopathy	4
Myelopathy	21
**mJOA score adjusted (0–10 points)**	
No myelopathy (10 points)	5
Slight myelopathy (8–9 points)	4
Moderate Myelopathy (6–7 points)	6
Severe myelopathy (0–5 points)	13
**Treatments**	39
Surgery	21
Endovascular	18

### Recurrence rate

Patients with DAVFs underwent a total of 29 procedures (Table 2) of which 16 surgical interventions and 13 endovascular treatments. At follow-up radiological examinations (MRA and/or spinal angiography) (8) all 16 patients who underwent surgery did not show disease recurrence, while in the endovascular treatment group six of them showed a recurrence (46%). These results reached a statistical significance (*p* < 0.004). In the EAVFs group 4 patients relapsed even more times, mainly after endovascular treatments, needing then a surgical procedure (Table 3). The patients who relapsed after endovascular treatment had been treated by embolization with various materials and in all cases the embolic material had occluded the afferent feeders, giving the impression of interrupting arteriovenous shunts. However, the patients have a late recurrence as embolic material had not flowed to the fistula site and the myeloradicular vein, thus allowing the recall of new arterial afferent and the persistence of the shunts. The surgical treatment guaranteed a lasting closure of the fistula as it allowed to disconnect the myeloradicular vein and the portion of the epidural plexus involved by the arterial feeders. This comparison did not reach a statistical significance.

**Table 2:** Recurrence rate: Dural AVF (number of treatment).

	Recurrence	No recurrence	Total
Surgery	0	16	16
Endovascular	6	7	13
Total	6	23	29
			*p* < 0.004

**Table 3:** Recurrence rate: Epidural AVF (number of treatment).

	Recurrence	No recurrence	Total
Surgery	1	4	5
Endovascular	4	1	5
Total	5	5	10
			*p* = 0.2

No patient presented major complications (meningitis, massive hemorrhages requiring evacuation, worsening of neurological deficit, significative vascular dissection) due to surgical and endovascular procedures.

### Clinical outcomes

Clinical outcomes were evaluated by studying the evolution of the symptoms at follow-up (at least 3 months). Most patients (21 cases) showed clinical improvement while in the other cases the symptoms remained stable. In one case a progression of myelopathy was sustained because of the persistence of the fistula after the first treatment, but after 3 months and a subsequent operation with complete closure a general improvement was recorded. 75% of the patients therefore had an improvement in symptoms following treatment. We haven't found correlation between the duration of symptoms before treatment and the possibility of obtaining improvement in symptoms. Outcomes follow-up was almost 6 months (range 3–8 months). We observed a correlation, albeit not very significant (Pearson's correlation index = 0.18), between the severity of myelopathy (mJOA) and the possibility of having an improvement in symptoms.

**Figure 3:**
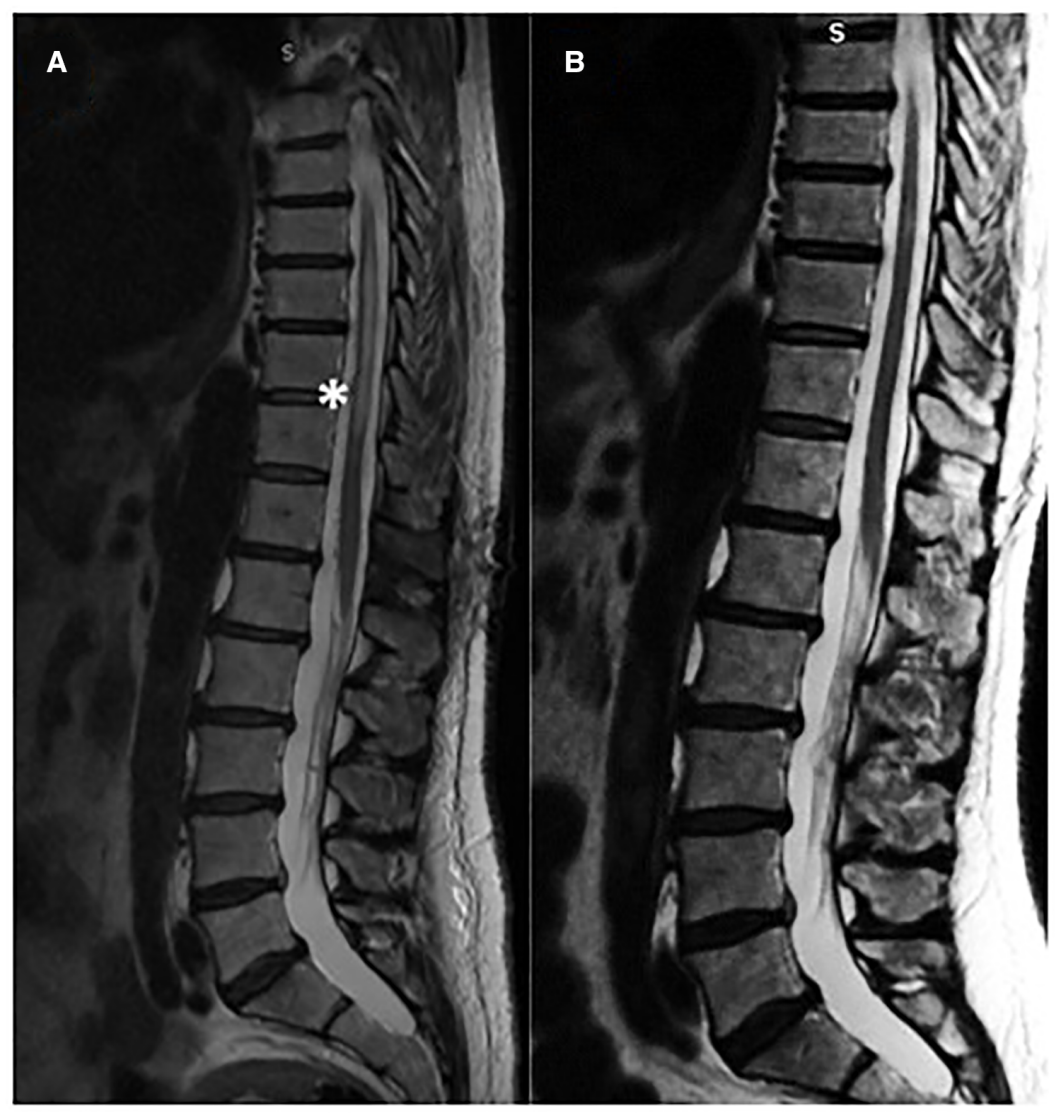
(**A**) Pre-operative MR showed myelopathy (**B**) post-operative good recovery of the myelopathy at three months follow-up.

## Discussion

The treatment strategy of spinal arteriovenous fistulas is based on the accurate localization of the fistula in the axial and sagittal planes. In the last decades our knowledge of these pathologies has increased and, as a result, different classification schemes have been proposed with the aim of better identifying the natural history, the clinical presentation, and the ideal treatment (3, 4). Spinal arteriovenous fistulas are currently believed to be a treatable cause of myelopathy (12, 13). There is therefore an indication to treat all spinal arteriovenous fistulas that are diagnosed. However, the neurological prognosis remains closely related to the severity of myelopathy and the duration of symptoms before treatment. In addition, clinical deterioration can also occur rapidly, so it is advisable to treat quickly once the diagnosis has been made. These types of malformations rarely could be associated with subarachnoid or subdural hemorrhage inside the spinal canal going into differential diagnosis with other vascular malformation (14, 15). Treatment consists in interrupting the shunt between the root artery and medullary vein whether it is in the epidural space or in the context of the dura mater (Figure 2). This can be done either surgically or endovascularly (16).

### Dural spinal arteriovenous fistula

Microsurgery is an important treatment option for most dural AV fistulas (5), despite advances in endovascular techniques. Surgery has the highest rate of complete occlusion and, therefore, is preferable for patients with advanced myelopathy to provide rapid and reliable elimination of the fistula (Figure 3). Surgical treatment is also the best option in situations in which superselective catheterization of the feeding artery reveals the contribution to the vascularization of the spinal cord as well, such as in cases in which the Adamkiewicz artery originates from the same radicular artery which supplies the fistula. In a comparative study Cesak T. et al. showed that surgical option for DAVF seems to be a more efficient treatment in terms of the clinical effect, occlusion and lower recurrence rate in comparison with the endovascular one (17).

### Epidural spinal arteriovenous fistula

Endovascular option is currently the preferred method of treatment for epidural arteriovenous fistulas, with approximately two-thirds of the studies in the literature reporting the use of endovascular treatment. Huang et al. (6) in their systematic review of the endovascular and surgical management of epidural AVFs, reported initial occlusion rates of 55% with endovascular treatment which increased to 92% with subsequent treatments. Trans-arterial embolization was the primary means of treatment in their series. Clinical improvement rates were 91% with endovascular treatment, with worsening of symptoms in only 3% of cases. With surgical treatment, Huang et al. reported complete occlusion rates of 74% on initial treatment and 87% at follow-up following subsequent interventions. Clinical improvement rates with surgery were 84%, and only 9% of patients reported worsening of symptoms. Endovascular treatment of EAVFs with intradural venous drainage focuses on occlusion of the epidural venous varix together with the proximal intradural draining vein. Failure to completely occlude the venous varix (shunt point) and draining vein may result in recanalization of the fistula due to the recruitment of additional arterial afferents. This is especially true for cervical fistulas. Trans-arterial embolization is often difficult to perform when the afferent arteries are small or tortuous. Surgical treatment is preferred in cervical fistulas with arterial afferents from the vertebral artery and in thoracic fistulas with afferents in common with the Adamkiewicz artery.

Microsurgical option is generally reserved for lesions that are difficult to treat endovascularly or after failure of endovascular treatment. The latter is often preferred when the fistula is close to the anterior spinal artery or is supplied by multiple arterial branches. An advantage of surgical treatment is the opportunity to perform both decompression and ligation, thus reducing the associated mass effect. This treatment strategy is particularly useful in lesions without intradural venous drainage. Surgical management is not always straightforward due to the often anterior location of these lesions which may require adequate exposure with a wider posterolateral approach. Bertonnier et al. (16) in a multicenter retrospective study evaluated neurological outcomes of 63 cases by analyzing the differences between patients undergoing surgical treatment and those undergoing endovascular treatment. No differences in terms of neurological outcome were found between patients who underwent surgery and those who underwent endovascular treatment. Subgroup analysis showed that patients who underwent surgery or embolization without recurrence experienced symptom improvement, while patients who underwent surgery or embolization with recurrence did not have symptom improvement. The initial occlusion rate was in favor of surgery, with 91.3% vs. 70% for endovascular treatment. The late recurrence rate was higher for embolization (21.4%) than for surgery (9.1%).

In a meta-analysis Byun et al. (18) analyzed a total of 123 patients treated for a thoracic or lumbosacral spinal epidural arteriovenous fistula. Endovascular treatment was performed in 67.5% of cases, surgical treatment in 23.6% and combined treatment in 8.9% of cases. The overall fistula closure rate was 83.5% and did not differ between groups. Clinical symptoms improved in 70.7% of patients, were stable in 25% and worsened in 1.7% with no difference between treatment modalities.

### Surgical or endovascular treatment?

In our series, 23 patients with spinal dural arteriovenous fistula underwent a total of 29 procedures. All 16 patients who underwent surgery had no recurrence at follow-up examinations performed 1 month after the operation and no clinical impairment at six months follow up. This trend match with literature of this topic (17, 19). In their meta-analysis Goyal et al. (20) report that with regard to dural fistulas, the recurrence rate and clinical outcomes are in favor of surgical treatment, although the new embolic materials (ONYX) have also improved the results of the endovascular options.

Of the 13 patients who underwent endovascular treatment by trans-arterial embolization with Onyx, 6 (46%) relapsed 1 month after treatment. The difference between the two treatments was statistically significant (*p* < 0.05). No major complications occurred following either surgical treatment or endovascular embolization. Surgical treatment has been shown to ensure more effective resolution of the fistula. Endovascular treatment, while not guaranteeing the same success rate, should be considered a less invasive technique which should be offered to selected patients with contra-indications for surgery (21). Regarding the 5 patients with EAVFs, a recurrence occurred in 20% of the surgical treatments and in 80% of the endovascular treatments. Given the small number of procedures, the difference between surgery and endovascular embolization is not to be held statistically significant (*p* > 0.05). Our study, even if statistically slightly significant, tend to agree with literature (22).

A Key point in the choice of treatment for this vascular malformation consists in the correct differential diagnosis between dural and epidural fistulas because of their similarity in pathological and clinical picture. After a proper diagnostic depiction, the best treatment should be individually selected.

**Figure 4:**
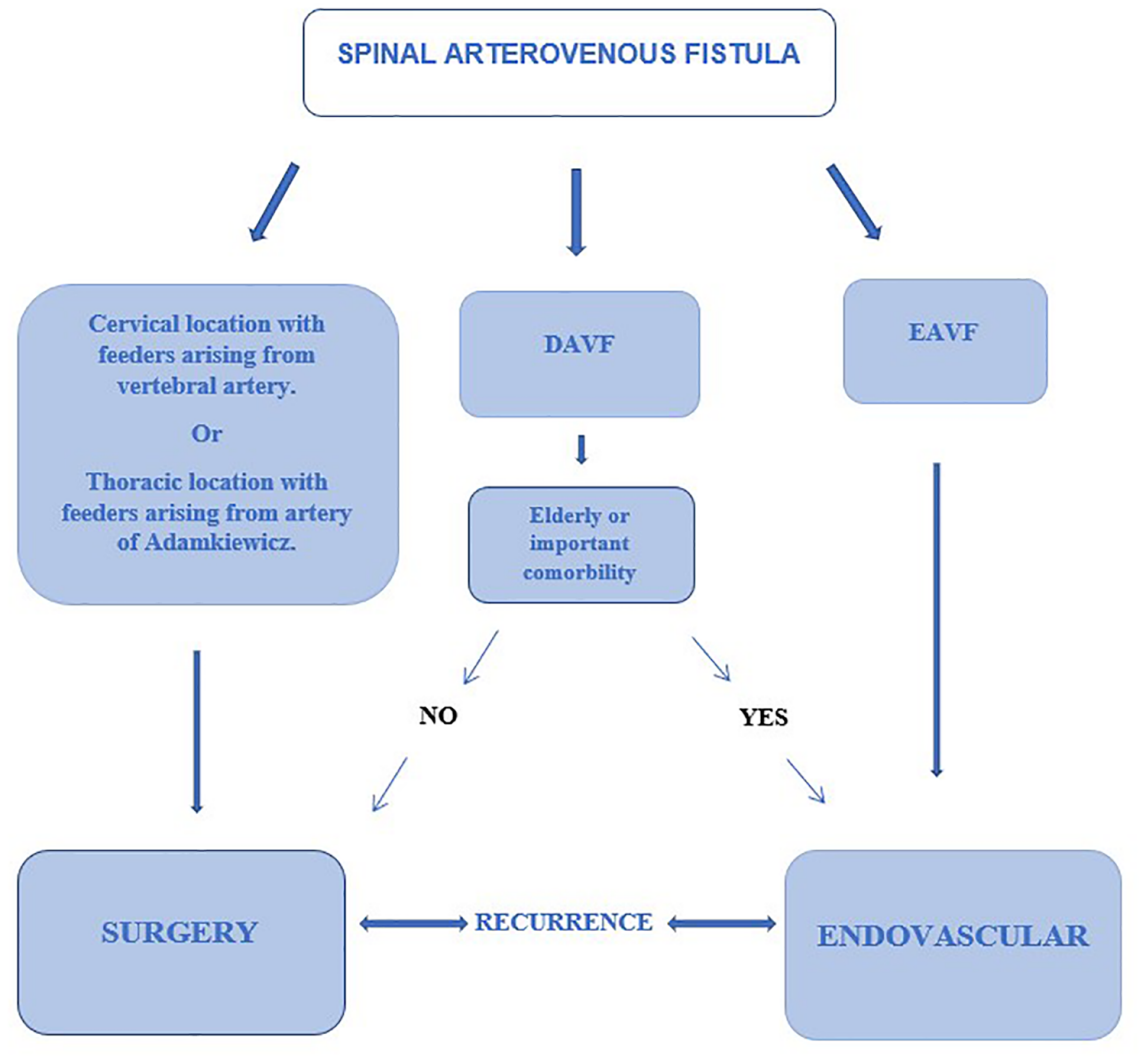
Decision making algorithm for treatment of spinal arteriovenous fistula.

As far as spinal epidural arteriovenous fistulas are concerned, we have seen how the site of the shunt is often found at the level of an epidural venous ectasia ventral to the dural sac; this implies that the surgical approach may require major destabilization (and then instrumentation) of vertebral structures to be able to safely manage the malformation. For this reason, we believe that an endovascular treatment could be the first choice for these patients without involving a significant increase in the risk of recurrence. In Figure 4 a decision algorithm is proposed basing on this manuscript considerations that can help during the decision-making process.

## Conclusions

Treatment options currently include closure *via* surgery or embolization *via* endovascular treatment. Surgical treatment can be proposed as first-line treatment for spinal dural arteriovenous fistulas. Endovascular embolization can be proposed in selected cases, as a less invasive technique, for elderly patients or with important comorbidities who could run greater risks undergoing surgery.

In spinal epidural arteriovenous fistulas, in view of the greater invasiveness of the surgical treatment and the non-significant difference in terms of recurrence risk between the two techniques, endovascular treatment can be proposed as a first line; in the event of a recurrence, a surgical intervention will instead be proposed in a short time.

## Limitation of study

The main limitation of the study could be the comparison analysis: two groups are heterogeneous and statistically incomparable in terms of clinical and morphological features. Moreover, the little samples size of our cohort, and short follow-up make statistical analysis slightly significative (especially for EAVF group).

## Data Availability

The original contributions presented in the study are included in the article/Supplementary Material, further inquiries can be directed to the corresponding authors.
